# Supplementary material for: A Transcriptome Community-and-Module Approach of the Human Mesoconnectome
Source: Entropy (Basel). 2021 Aug 11;23(8):1031. doi: 10.3390/e23081031 (PMC8393183; doi:10.3390/e23081031)
Supplement: Supplementary file 1 [file entropy-23-01031-s001.zip › entropy-1291561-supplementary/Entropy_Data_in_Brief.pdf]

Gene Ontology (GO) word clouds

To build the GO word clouds, we extracted the p values of each community for the ontologies found using ViSeAGO. We filtered out all entries with a corrected p-value <0.05, all remaining significance values were considered as 1. Then, we calculated the p-value base 10 logarithm and multiplied it by -1 to manipulate positive numbers.

Afterward, to retrieve only those GO entries unique to a community and thereby identify each community's biological role, we performed a uniform distribution test. Such test was a Kolmogorov-Smirnoff (KS) test for uniform distributions. Entries were considered as unique for each community if KS had a significance value lower than 0.01 and such GO was assigned to the community having the highest transformed p-value.

Then, for each community, we mined the GO tag names to perform a word distribution analysis, discarding articles, conjunctions to remove overabundant words and words such as "biological", "process", "molecular", "function", "cell" and "component", "regulation", "activity", "negative", "positive" to prevent labels associated with broader biological functions in the Gene Ontology. The filtered word distributions were used to generate the GO word clouds associated with each community display in supplementary figures Supplementary Figure S5-Supplementary Figure 17.

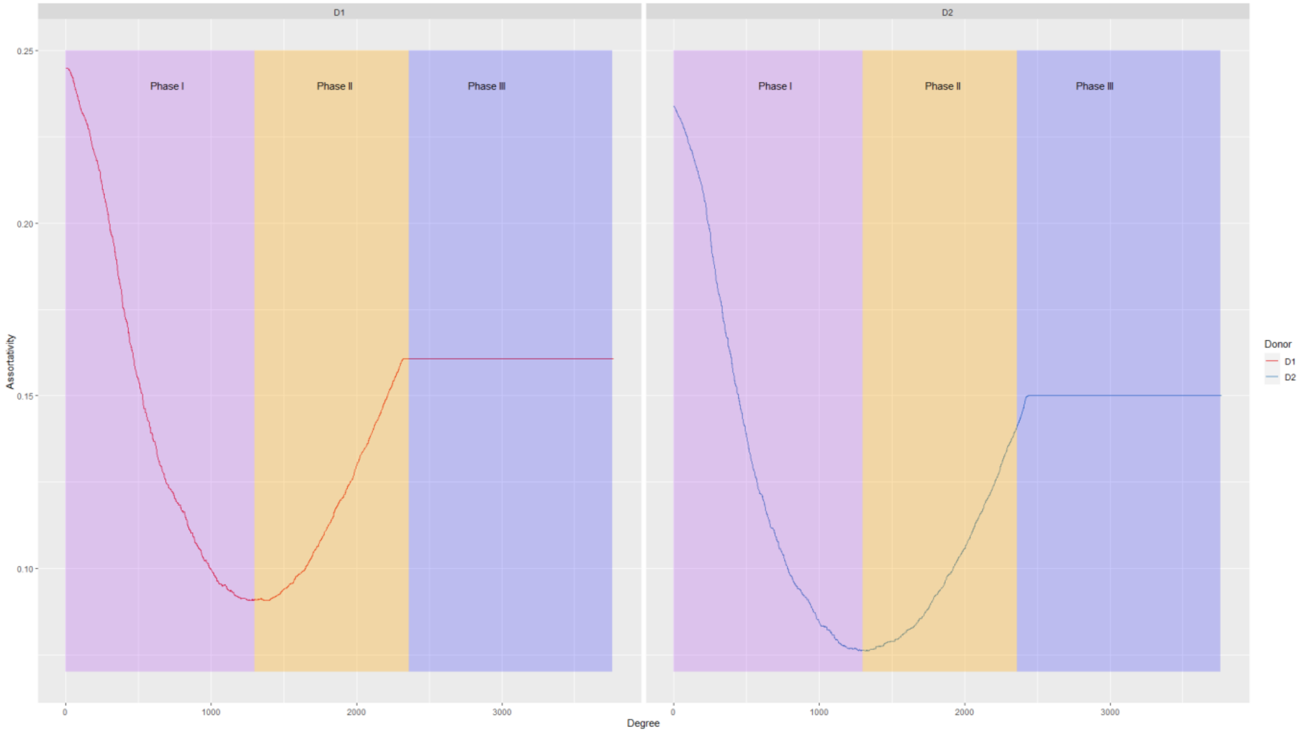

Supplementary Figure S1 Degree assortativity, in the left for D1 and in the right for D2. The degree assortativity shows a three-phase behavior corresponding to Phase I, an initial disassortative trend, Phase II, an assortative trend and, Phase III, a final neutral trend.

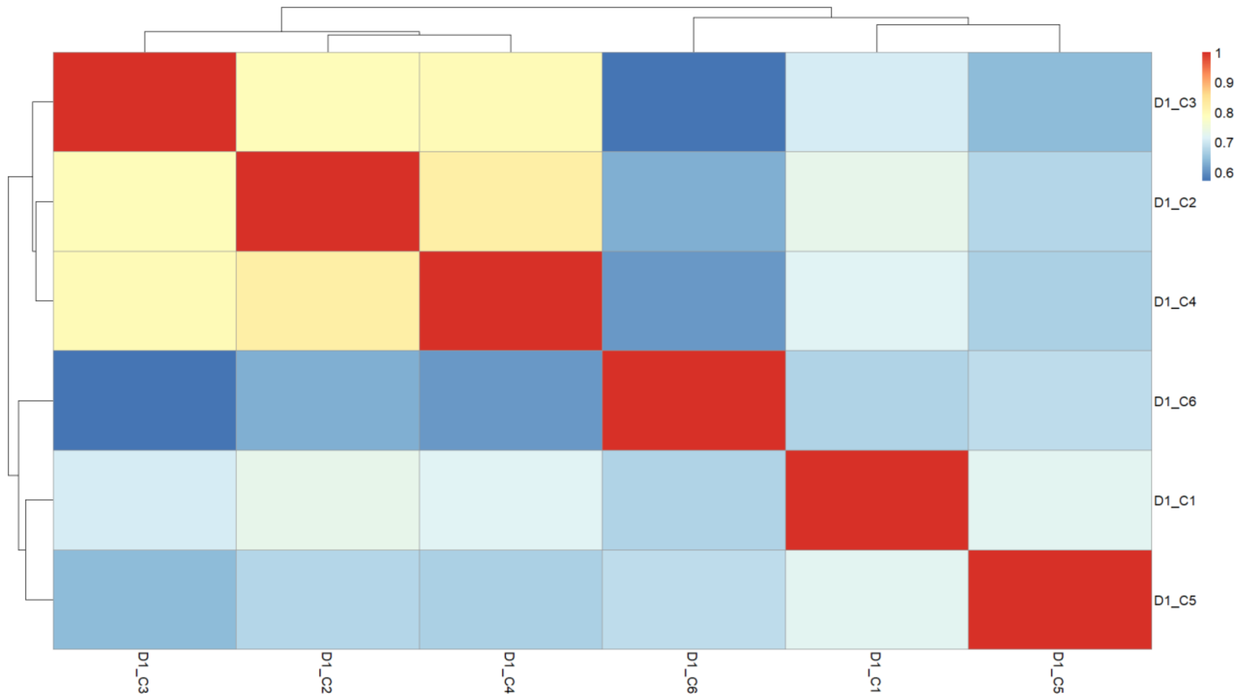

Supplementary Figure S2 Wang's Semantic Similarity for GOs entries in D1 communities.

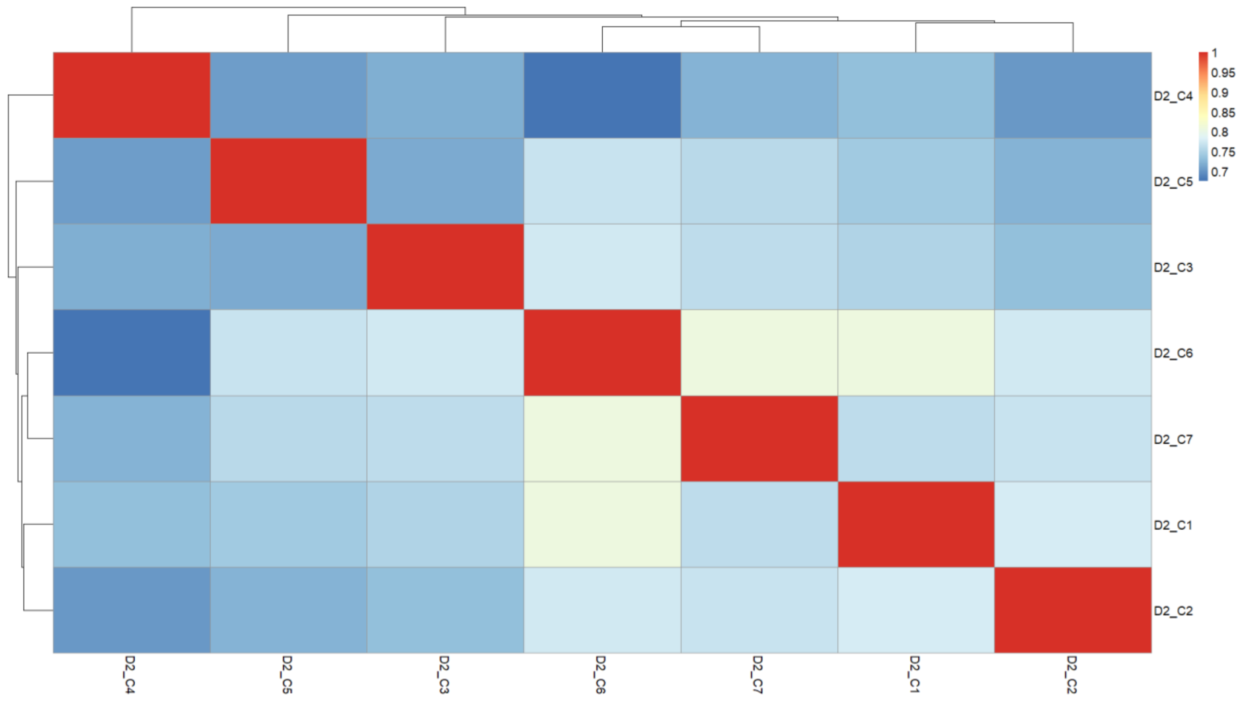

Supplementary Figure S3 Wang's Semantic Similarity for GOs entries in D2 communities.

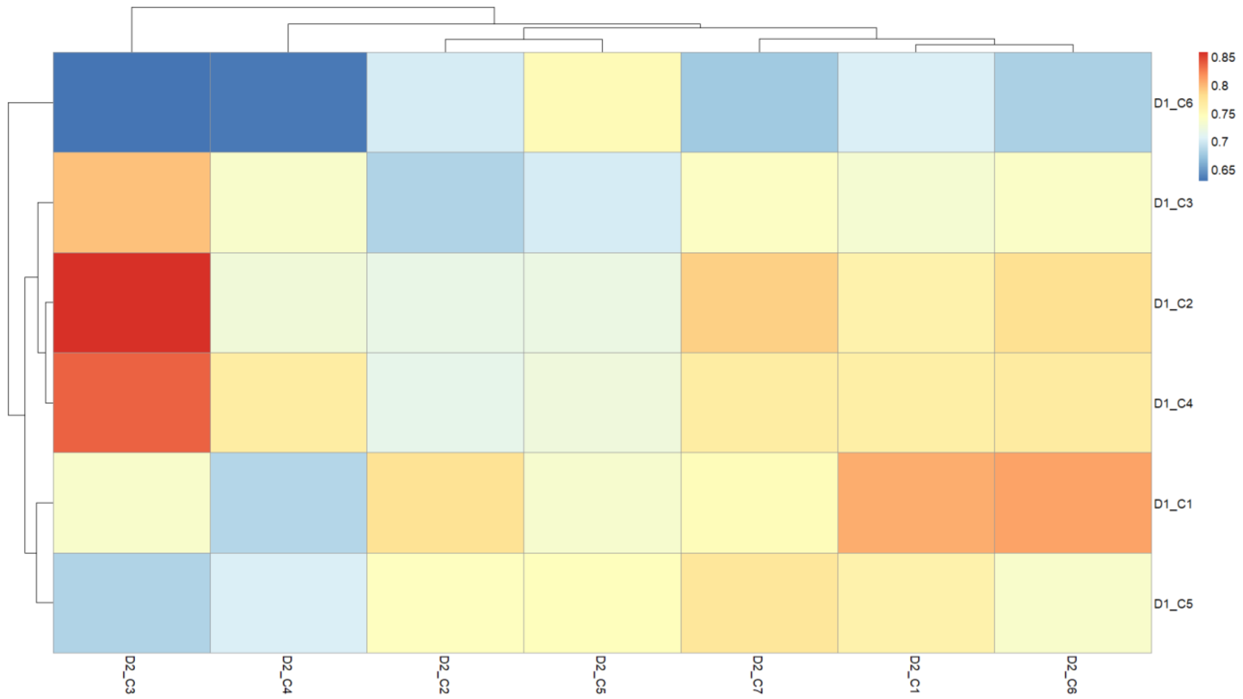

Supplementary Figure S4 Wang's Semantic Similarity for GOs entries in x-axis D1 communities and in y-axis D2 communities.

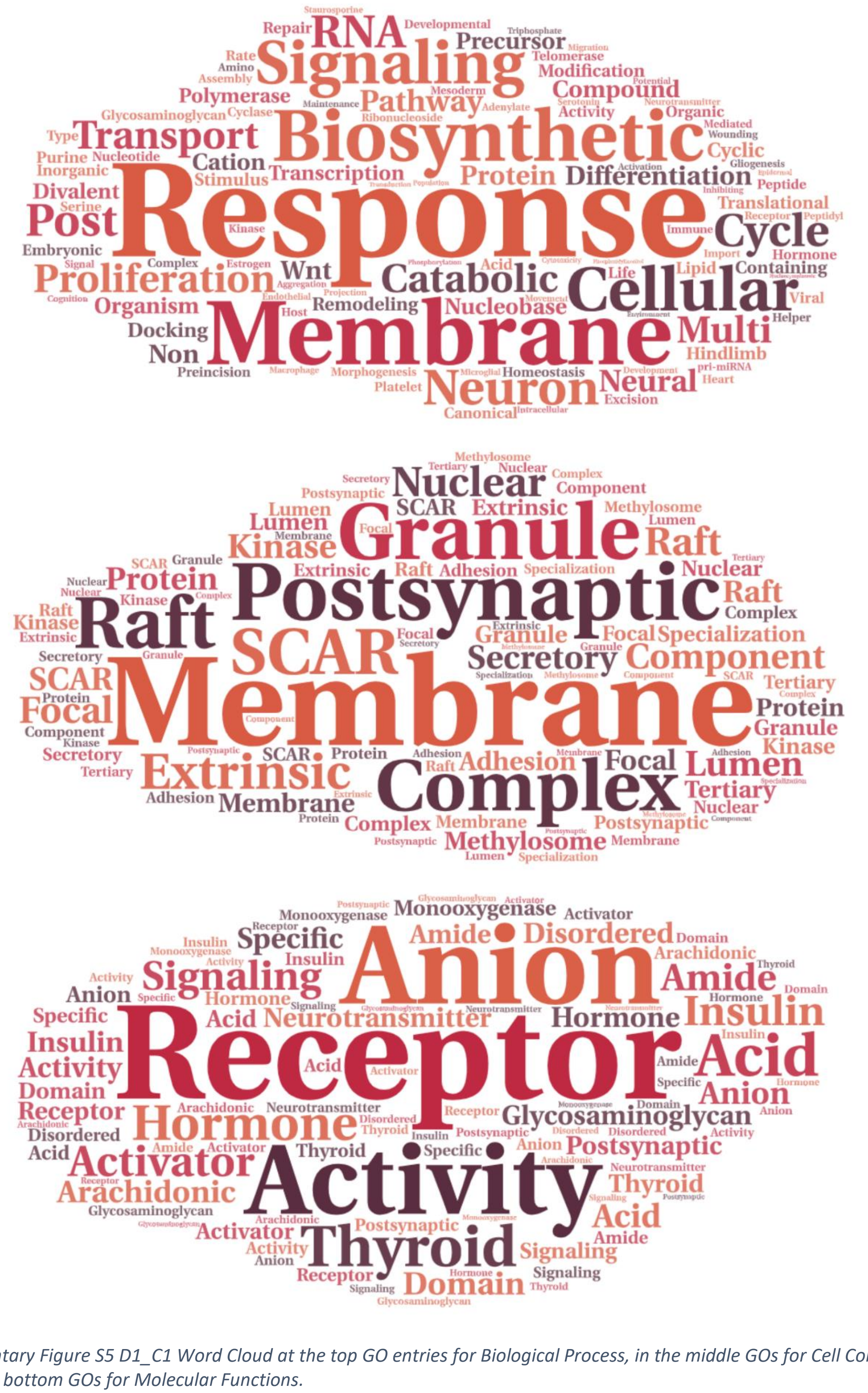

Supplementary Figure S5 D1_C1 Word Cloud at the top GO entries for Biological Process, in the middle GOs for Cell Component and, in the bottom GOs for Molecular Functions.

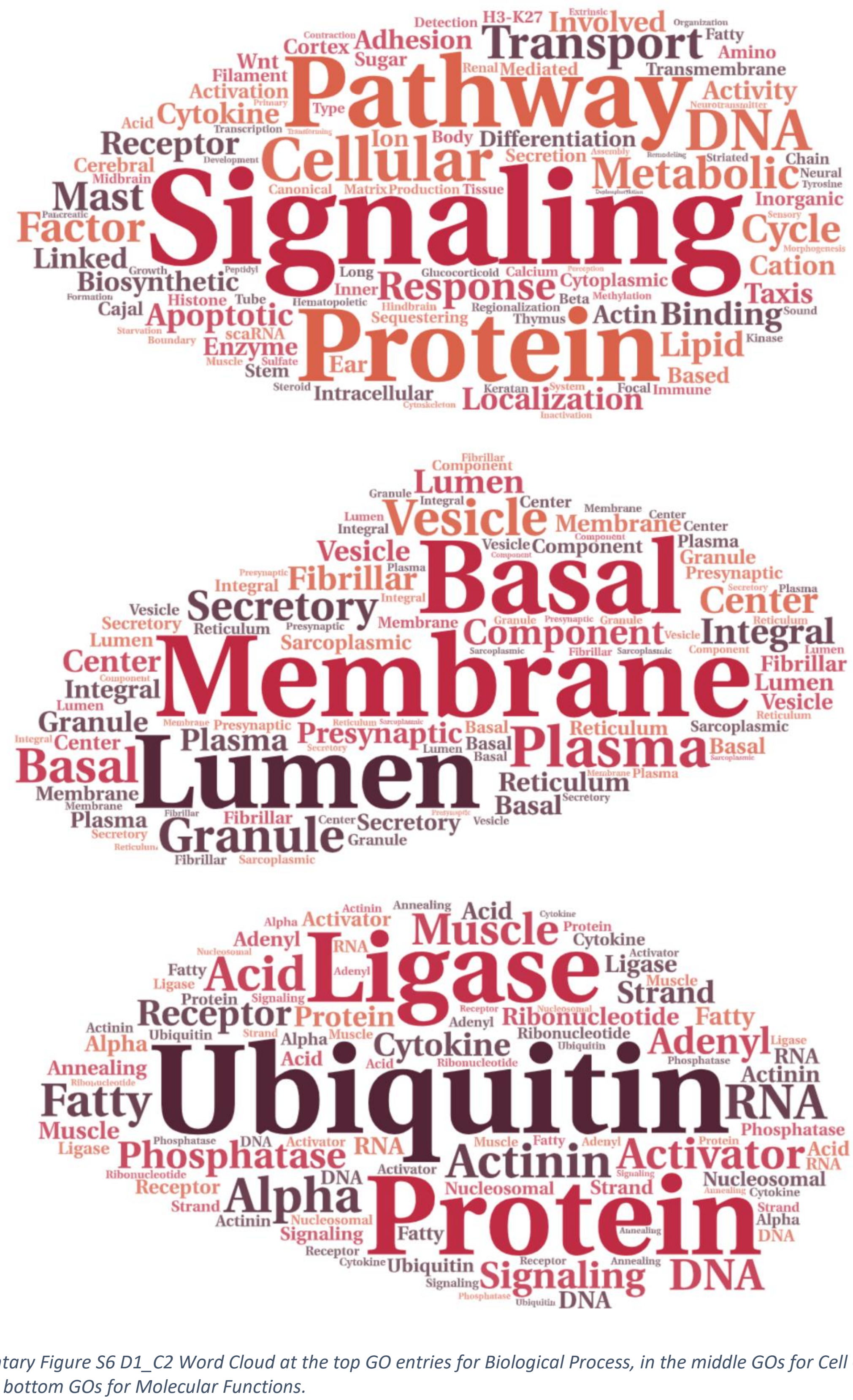

Supplementary Figure S6 D1_C2 Word Cloud at the top GO entries for Biological Process, in the middle GOs for Cell Component and, in the bottom GOs for Molecular Functions.

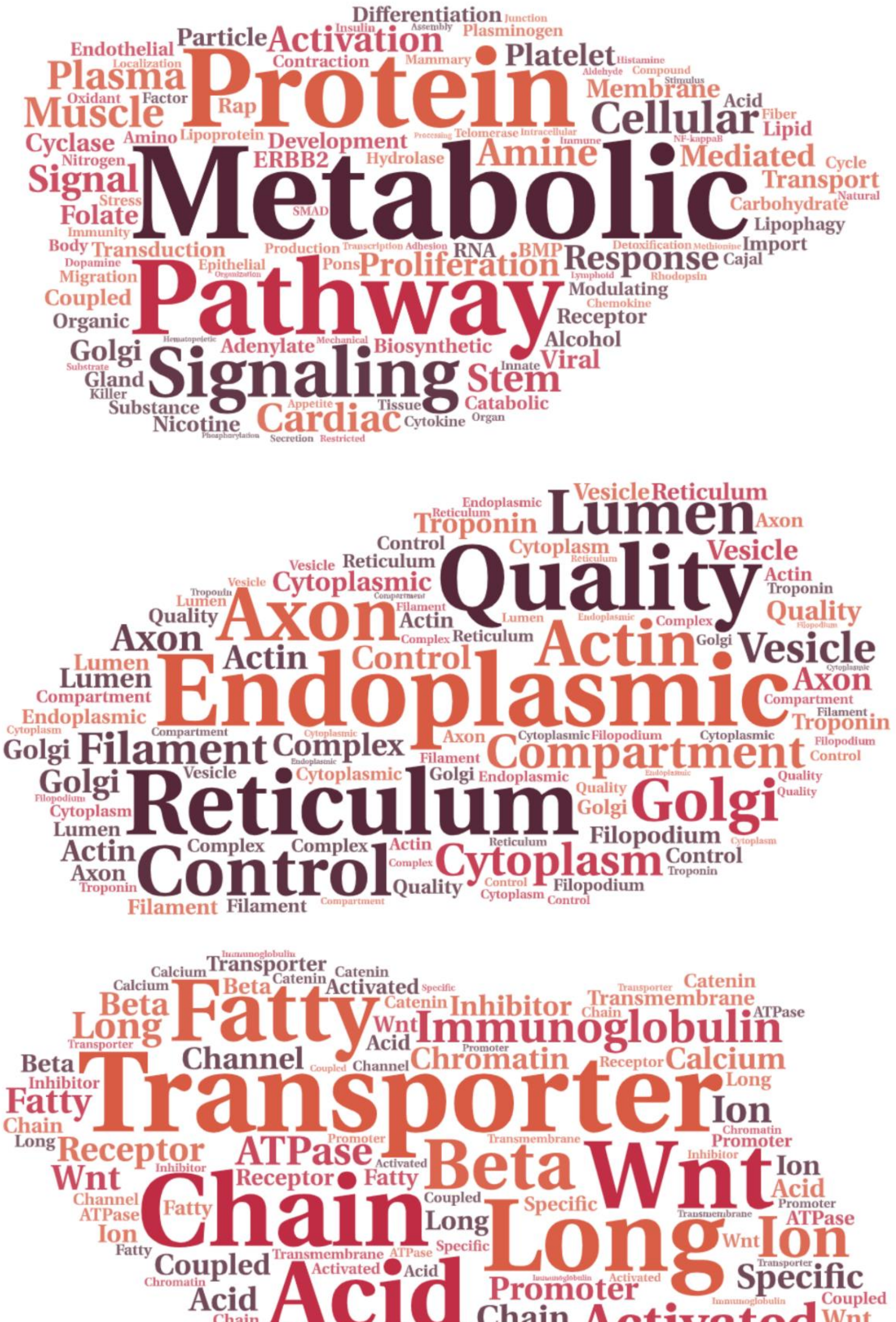

Supplementary Figure S7 D1_C3 Word Cloud at the top GO entries for Biological Process, in the middle GOs for Cell Component and, in the bottom GOs for Molecular Functions.

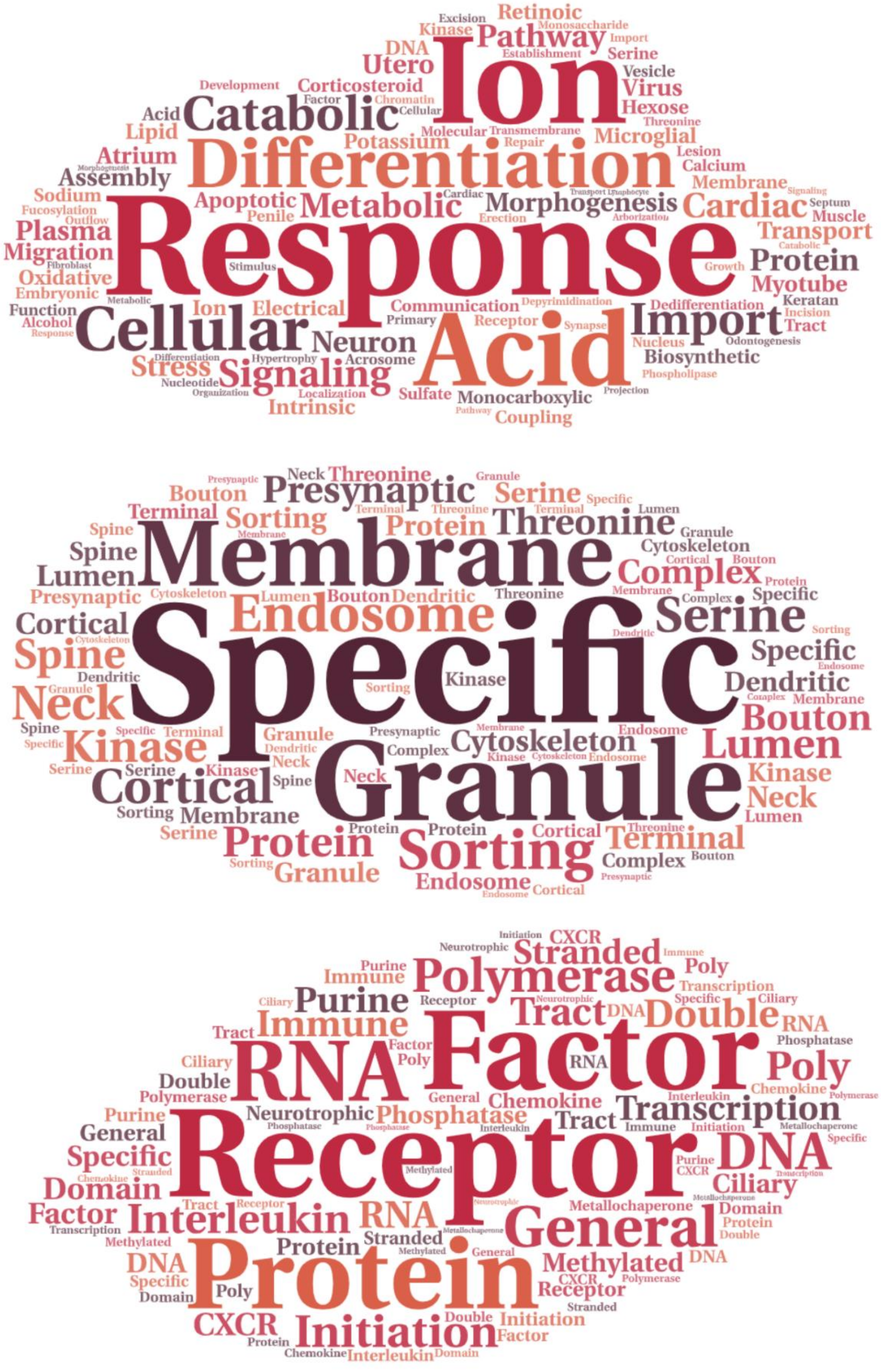

Supplementary Figure S8 D1_C4 Word Cloud at the top GO entries for Biological Process, in the middle GOs for Cell Component and, in the bottom GOs for Molecular Functions.

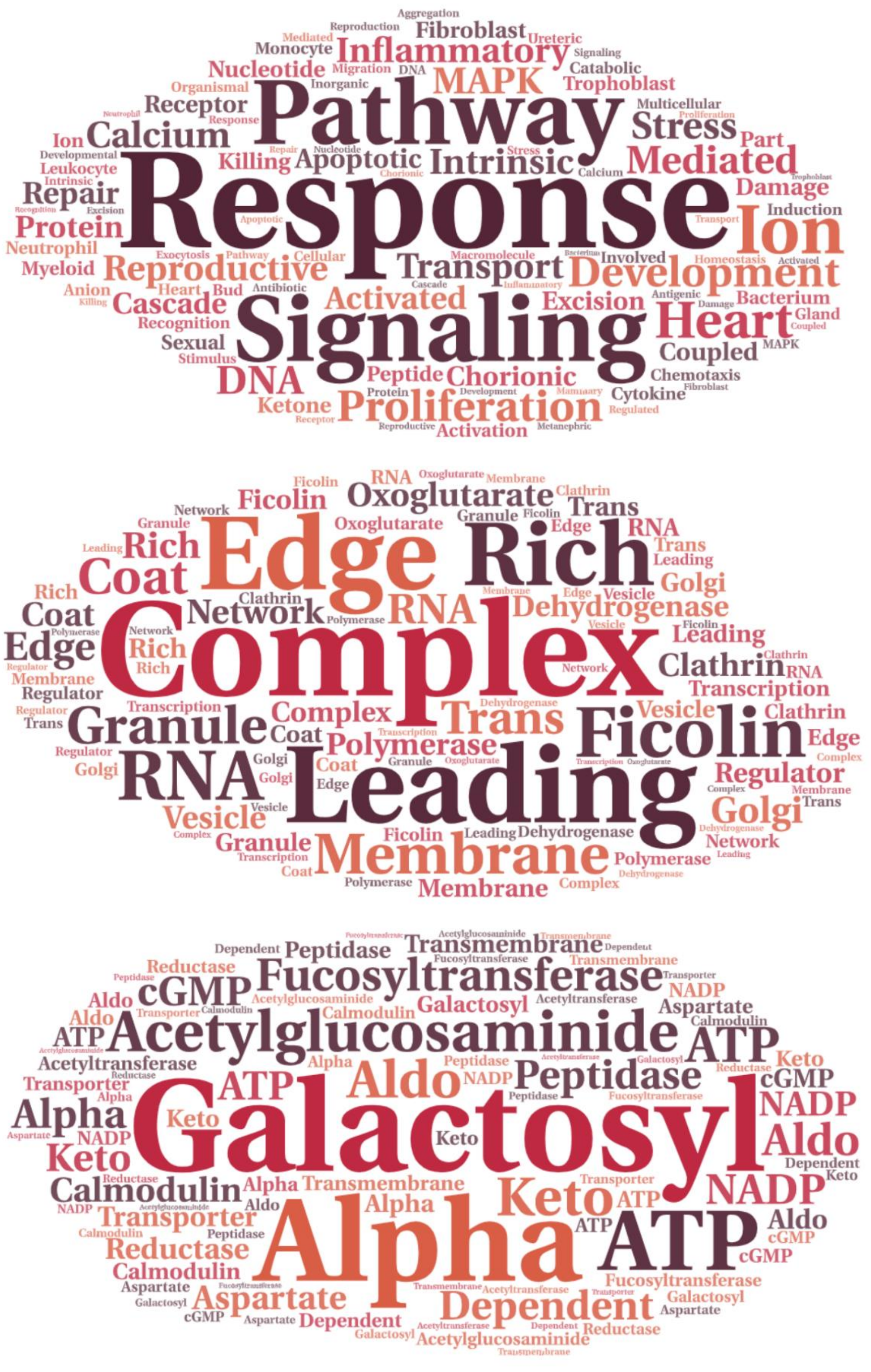

Supplementary Figure S9 D1_C5 Word Cloud at the top GO entries for Biological Process, in the middle GOs for Cell Component and, in the bottom GOs for Molecular Functions.

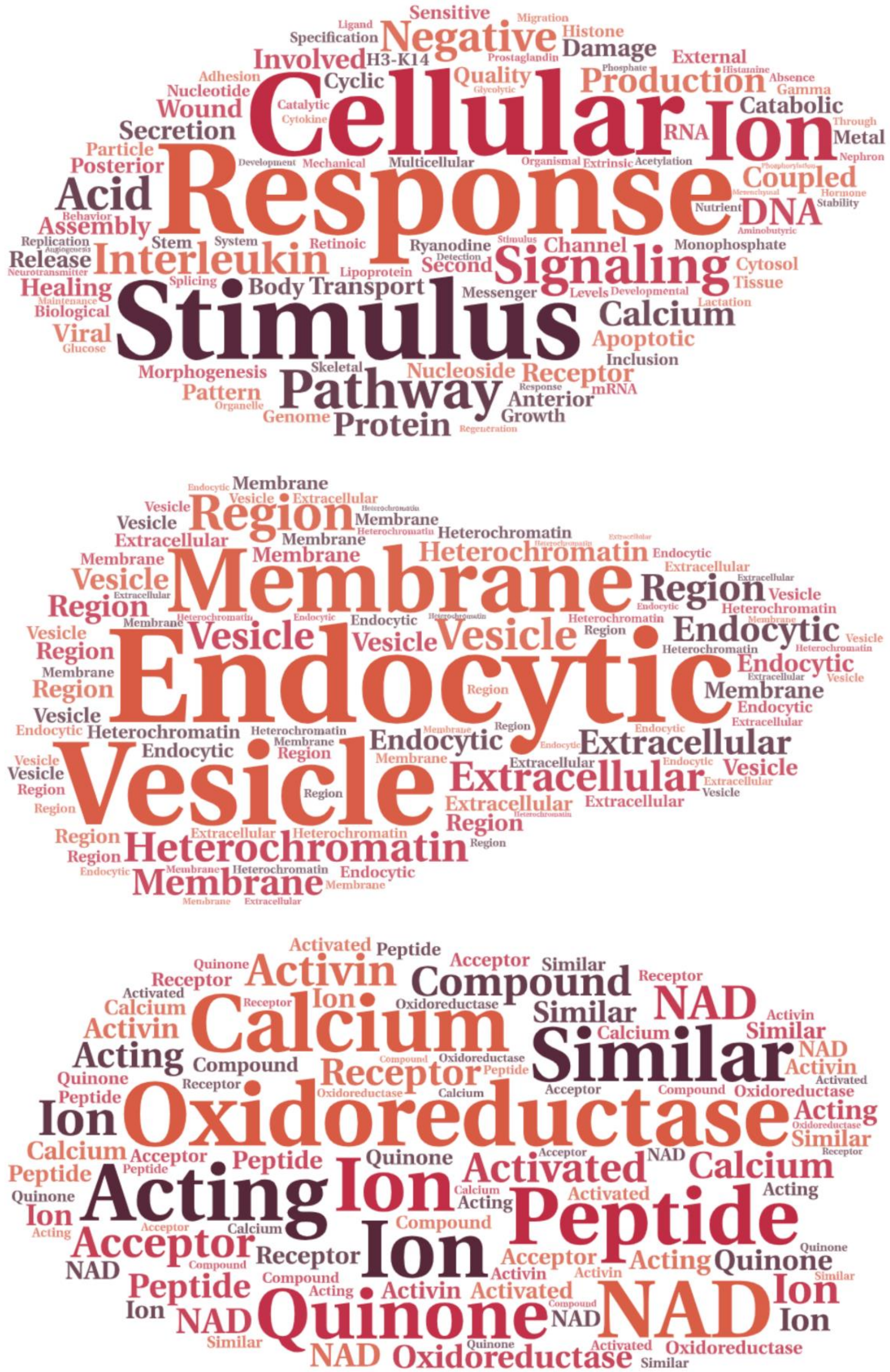

Supplementary Figure S10 D1_C6 Word Cloud at the top GO entries for Biological Process, in the middle GOs for Cell Component and, in the bottom GOs for Molecular Functions.

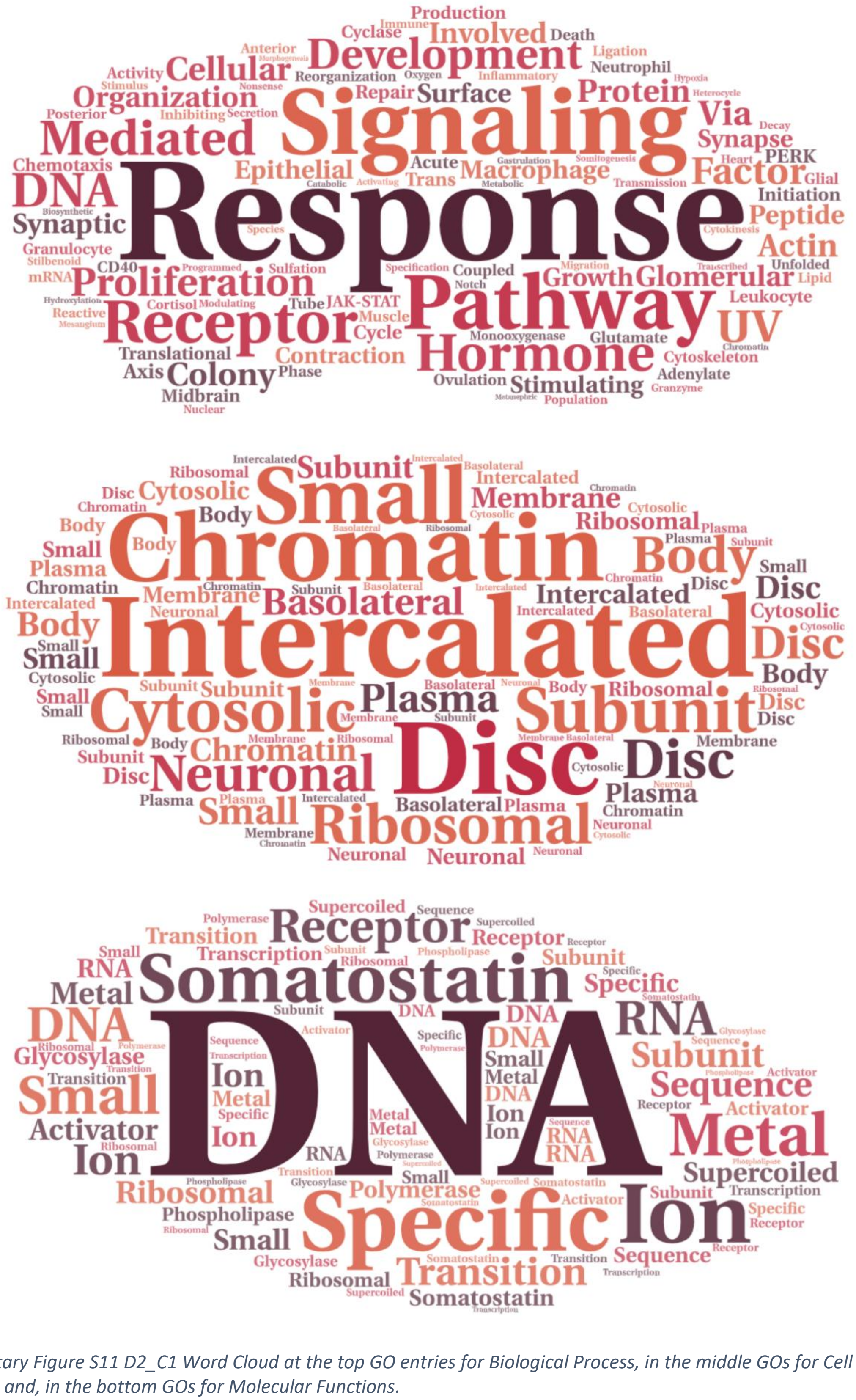

Supplementary Figure S11 D2_C1 Word Cloud at the top GO entries for Biological Process, in the middle GOs for Cell Component and, in the bottom GOs for Molecular Functions.

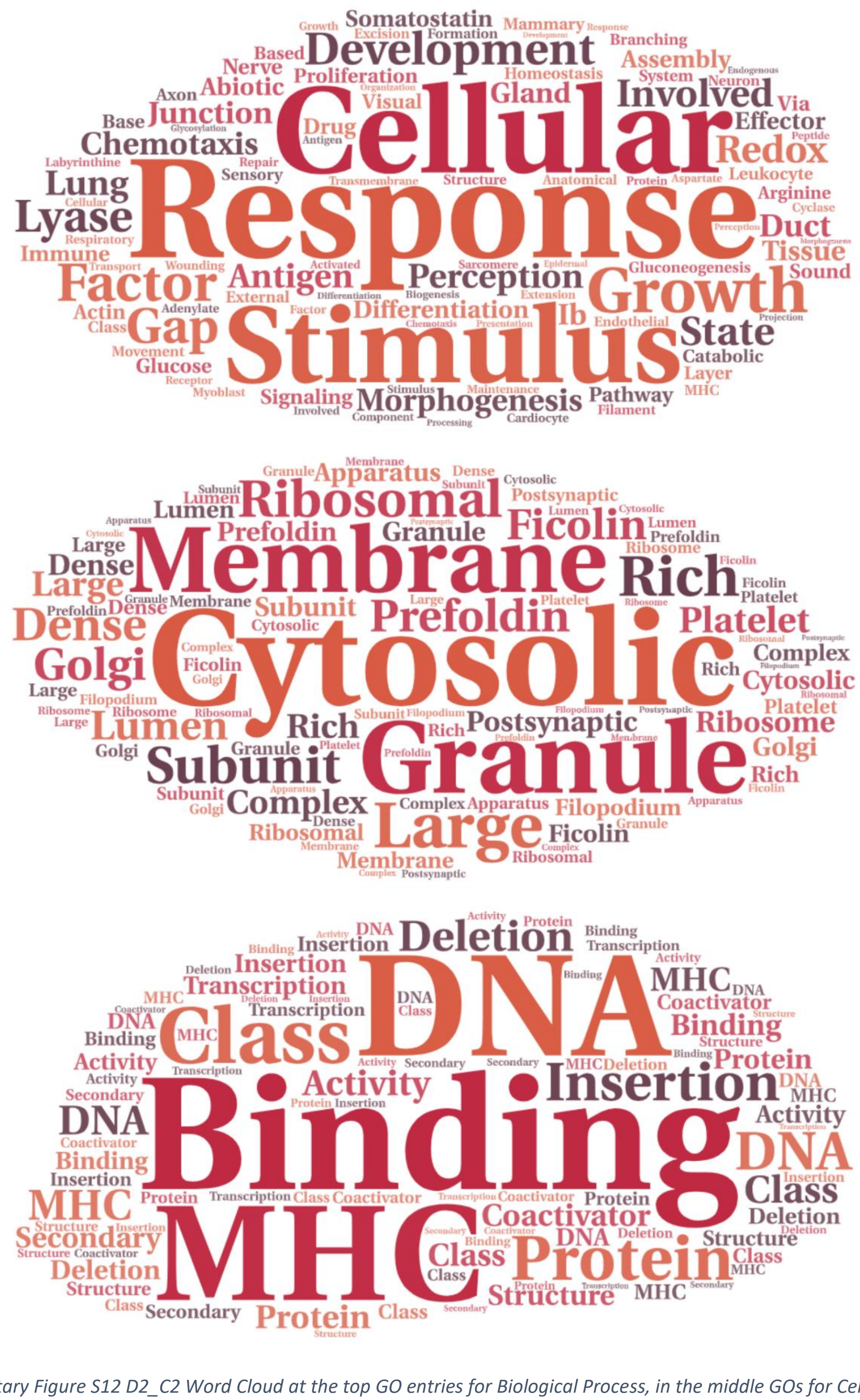

Supplementary Figure S12 D2_C2 Word Cloud at the top GO entries for Biological Process, in the middle GOs for Cell Component and, in the bottom GOs for Molecular Functions.

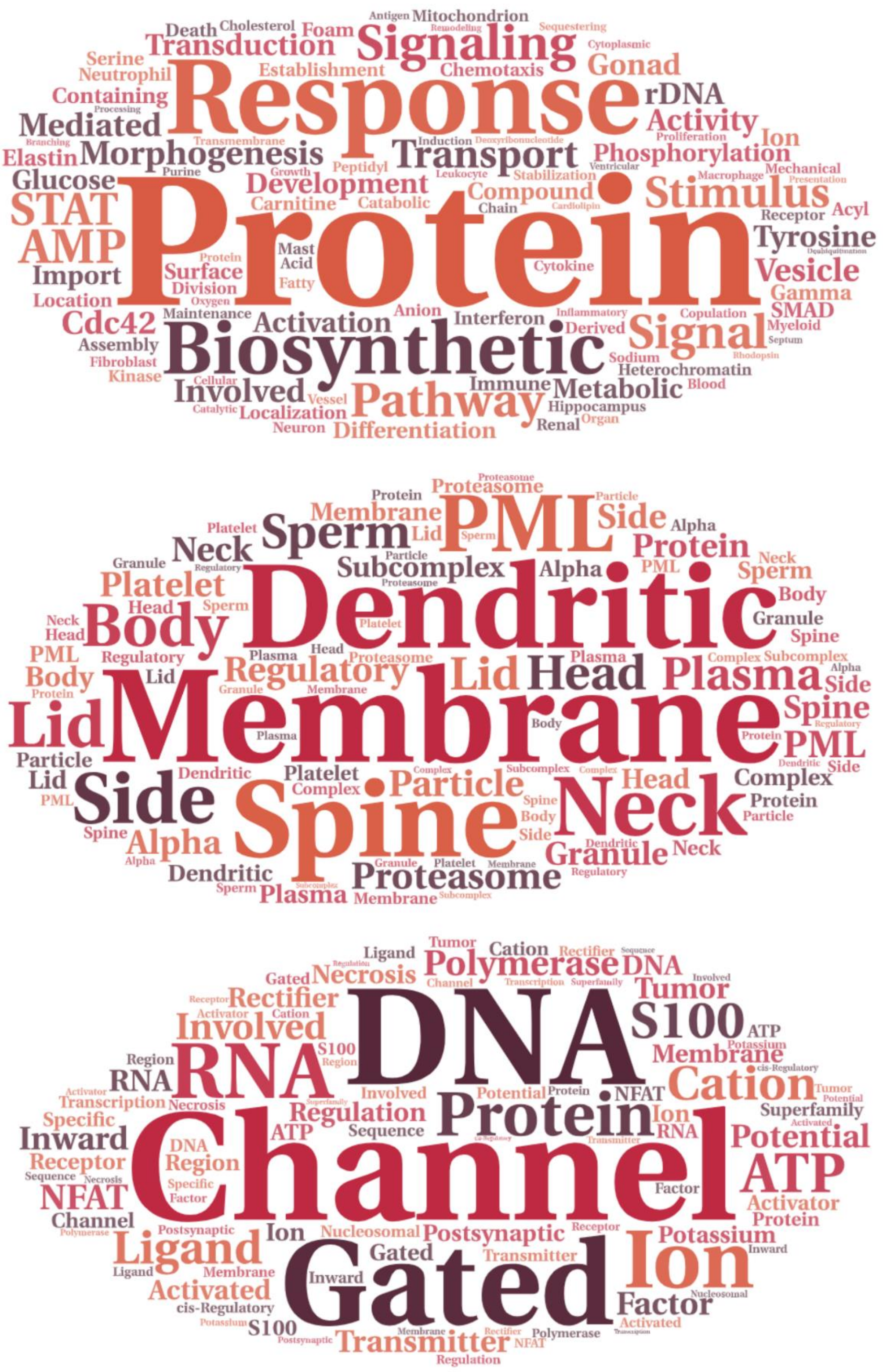

Supplementary Figure S13 D2_C3 Word Cloud at the top GO entries for Biological Process, in the middle GOs for Cell Component and, in the bottom GOs for Molecular Functions.

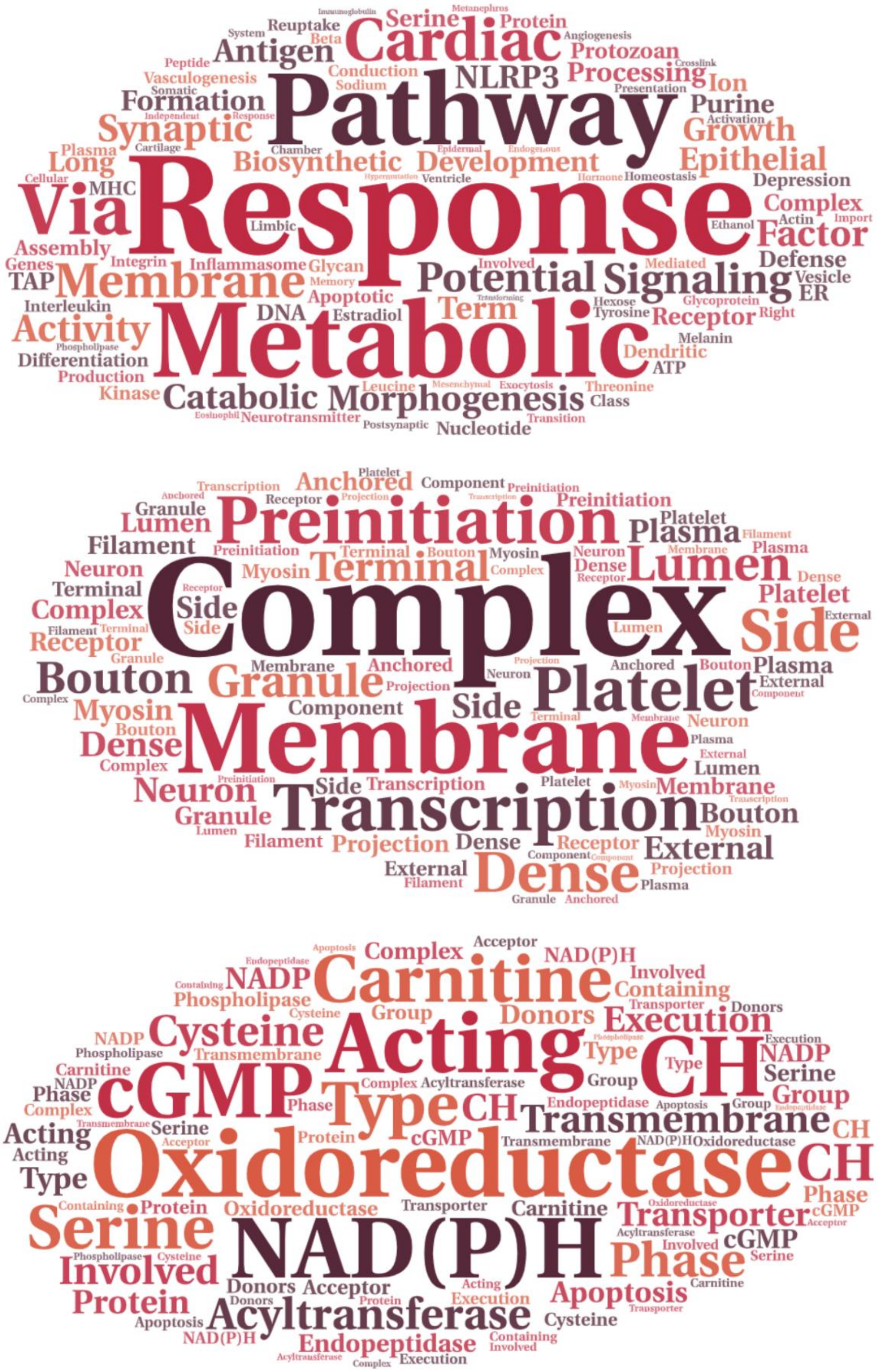

Supplementary Figure S14 D2_C4 Word Cloud at the top GO entries for Biological Process, in the middle GOs for Cell Component and, in the bottom GOs for Molecular Functions.

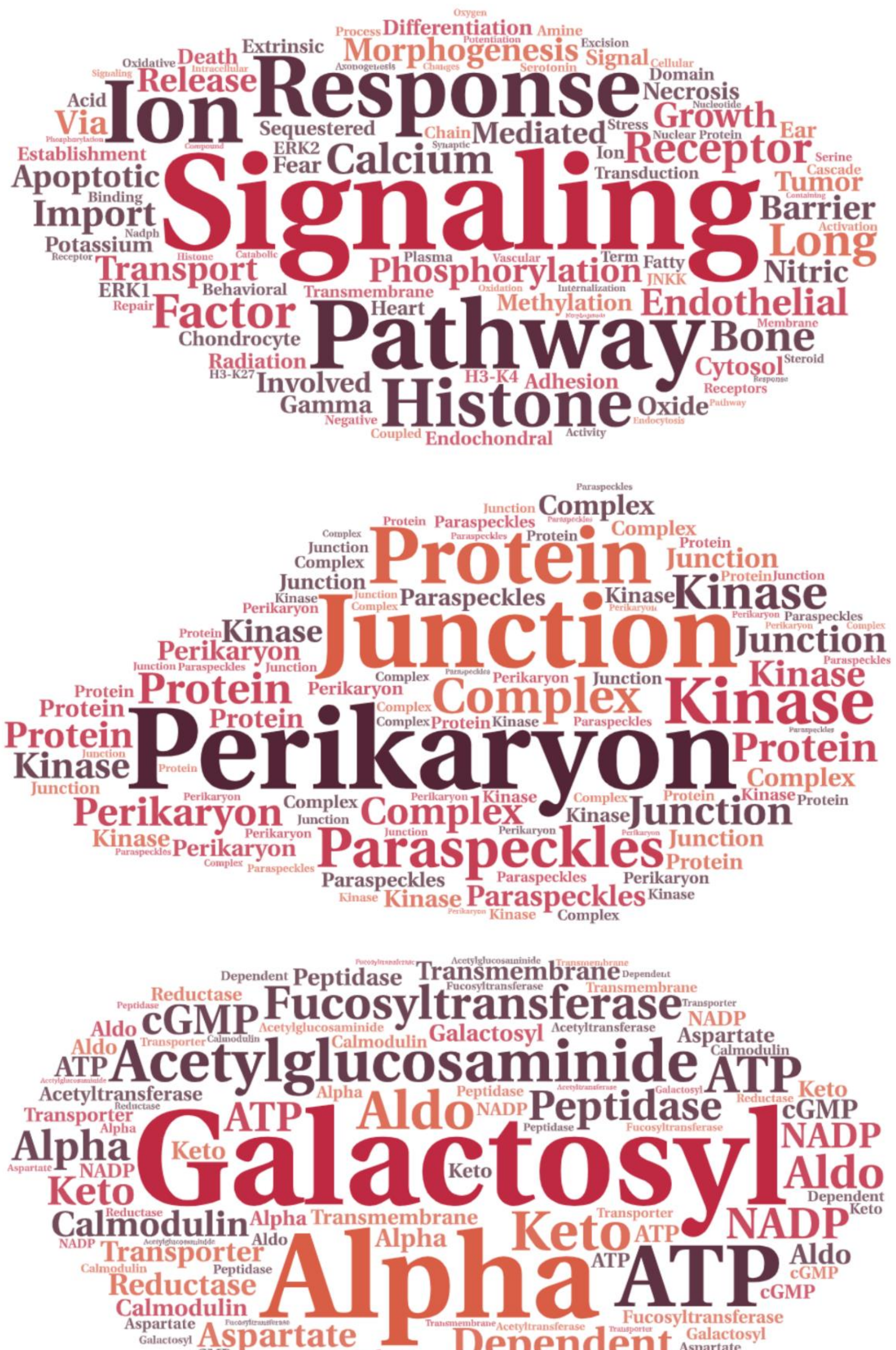

Supplementary Figure S15 D2_C5 Word Cloud at the top GO entries for Biological Process, in the middle GOs for Cell Component and, in the bottom GOs for Molecular Functions.

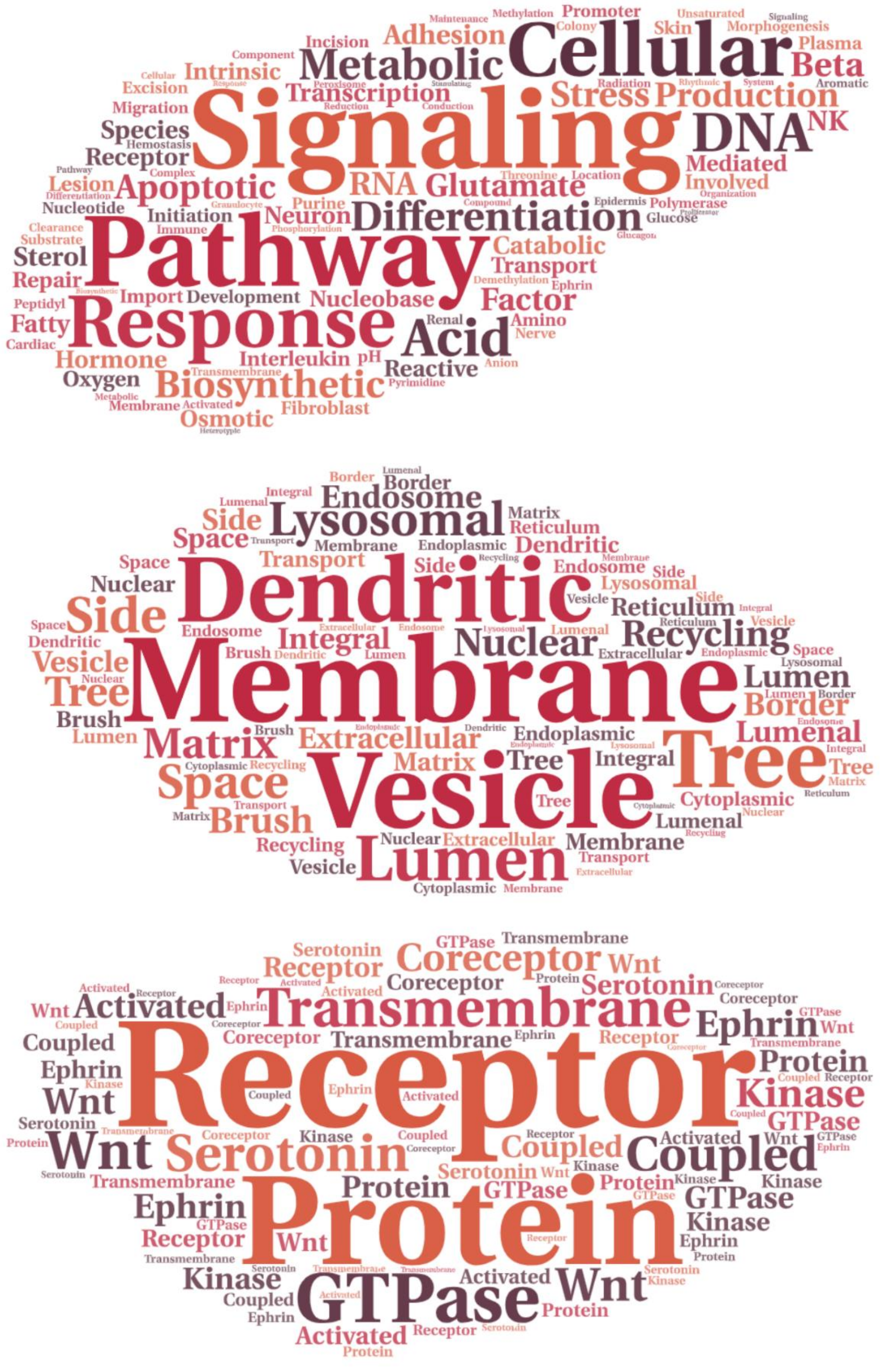

Supplementary Figure S16 D2_C6 Word Cloud at the top GO entries for Biological Process, in the middle GOs for Cell Component and, in the bottom GOs for Molecular Functions.

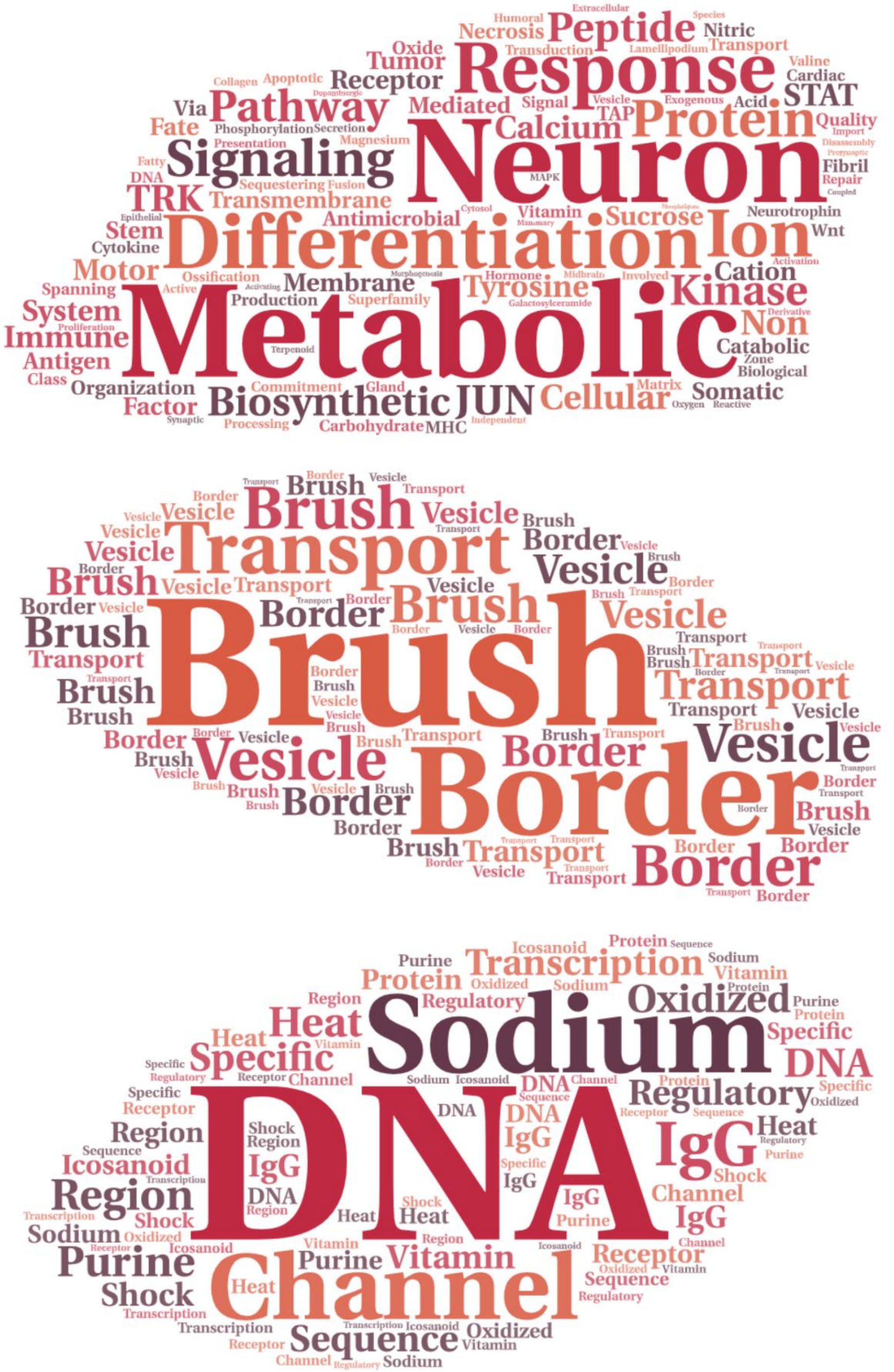

Supplementary Figure S17 D2_C7 Word Cloud at the top GO entries for Biological Process, in the middle GOs for Cell Component and, in the bottom GOs for Molecular Functions.

Abbreviation	Name	Abbreviation	Name
**PoG.il**	Inferior lateral postcentral gyrus	Pu	Putamen
**SPL.i**	Inferior bank of superior parietal lobule	GRe	Gyrus rectus
**He.VI**	Cerebellum lateral hemisphere VI	Pcu.i	Inferior lateral bank of precuneus
**AnG.i**	Inferior bank of angular gyrus	SFG.m	Medial bank of superior frontal gyrus
**HCd**	Head of caudate nucleus	AnG.s	Superior bank of angular gyrus
**PCLa.i**	Anterior part of paracentral lobule inferior bank	CgGf.s	Superior bank of cingulate gyrus
**orIFG**	Orbital part of inferior frontal gyrus	FuG.its	Interior sulcus of fusiform gyrus
**GPi**	Internal segment of globus pallidus	MTG.s	Superior bank of middle temporal gyrus
**He.Crus.I**	Cerebellum lateral hemisphere Crus I	CgGp.s	Superior bank parietal part of cingulate gyrus
**SPL.s**	Superior bank of superior parietal lobule	LiG.str	Striate of lingual gyrus
**BCd**	Body of caudate nucleus	PoG.cs	Central sulcus of postcentral gyrus
**SMG.i**	Inferior bank of supramarginal gyrus	Cun.pest	Peristriate of cuneus
**MFG.i**	Inferior bank of middle frontal gyrus	MOrG	Medial orbital gyrus
**LOrG**	Lateral orbital gyrus	Pcu.s	Superior lateral bank of precuneus
**PHG.cos**	Collateral sulcus of parahippocampal gyrus	He.VIIIA	Cerebellum lateral hemisphere VIIIA
**PoG.sl**	Superior lateral postcentral gyrus	STG.l	Lateral bank of superior temporal gyrus
**MFG.s**	Superior bank of middle frontal gyrus	STG.i	Inferior bank of superior temporal gyrus
**CgGf.i**	Inferior bank of cingulate gyrus	PV.IX	Cerebellum paravermis IX
**LiG.pest**	Peristriate lingual gyrus	PrG.prc	Precentral sulcus of precentral gyrus
**LIG**	Long insular gyri	PCLa.s	Superior bank of paracentral lobule
**PrG.sl**	Superior laterlar precentral gyrus	ITG.l	Lateral bank of inferior temporal gyrus
**MTG.i**	Inferior bank middle temporal gyrus	CgGp.i	Inferior bank of cingulate gyrus
**ITG.its**	Inferior sulcus of inferior temporal gyrus	PHG.l	Lateral bank of parahippocampal gyrus
**GPe**	External segment of globus pallidus	SFG.l	Lateral bank of superior frontal gyrus
**SIG**	Short insular gyri	PrG.il	Inferior lateral aspect of precentral gyrus

Supplementary Table S1 Allen Brain regions and its abbreviatures.
