# Supplementary material for: A Transcriptome Community-and-Module Approach of the Human Mesoconnectome
Source: Entropy (Basel). 2021 Aug 11;23(8):1031. doi: 10.3390/e23081031 (PMC8393183; doi:10.3390/e23081031)
Supplement: Supplementary file 1 [file entropy-23-01031-s001.zip › entropy-1291561-supplementary/Supplementary Figure S24.html]

Chord Diagram

Download
