# Supplementary material for: A Transcriptome Community-and-Module Approach of the Human Mesoconnectome
Source: Entropy (Basel). 2021 Aug 11;23(8):1031. doi: 10.3390/e23081031 (PMC8393183; doi:10.3390/e23081031)
Supplement: Supplementary file 1 [file entropy-23-01031-s001.zip › entropy-1291561-supplementary/Supplementary Files.pdf]

Supplementary Files

Entropy_Data_in_Brief

Methodology for ontology word clouds, Supplementary Figures S1-S17 and Supplementary Table S1.

Supplementary Table S2

D1 ontologies for non-variant genes.

Supplementary Table S3

D2 ontologies for non-variant genes.

Supplementary Table S4

D1 ontologies for variant genes.

Supplementary Table S5

D2 ontologies for variant genes.

Supplementary Table S6

Gene list for both donor communities.

Supplementary Table S7

Modules of brain areas for the D1.

Supplementary Table S8

Modules of brain areas for the D2.

Supplementary Figure S18

Heatmap of p-value for Biological Process GOs for D1.

Supplementary Figure S19

Heatmap of p-value for Cellular Component GOs for D1.

Supplementary Figure S20

Heatmap of p-value for Molecular Function GOs for D1.

Supplementary Figure S21

Heatmap of p-value for Biological Process GOs for D2.

Supplementary Figure S22

Heatmap of p-value for Cellular Component GOs for D2.

Supplementary Figure S23

Heatmap of p-value for Molecular Function GOs for D2.

Supplementary Figure S24

Brain connectivity for D1 brain areas through modules.

Supplementary Figure S25

Brain connectivity for D2 brain areas through modules.
